# The Use of Information and Communication Technologies by Sex Workers to Manage Occupational Health and Safety: Scoping Review

**DOI:** 10.2196/26085

**Published:** 2021-06-24

**Authors:** Thérèse Bernier, Amika Shah, Lori E Ross, Carmen H Logie, Emily Seto

**Affiliations:** 1 Institute of Health Policy, Management and Evaluation Dalla Lana School of Public Health University of Toronto Toronto, ON Canada; 2 Dalla Lana School of Public Health University of Toronto Toronto, ON Canada; 3 Factor-Inwentash Faculty of Social Work University of Toronto Toronto, ON Canada; 4 Women’s College Research Institute Women’s College Hospital Toronto, ON Canada; 5 Centre for Gender and Sexual Health Equity Vancouver, BC Canada

**Keywords:** sex work, smartphone, mobile phone, occupational health and safety, online, internet, website

## Abstract

**Background:**

In many countries, sex work is criminalized, driving sex work underground and leaving sex workers vulnerable to a number of occupational health and safety risks, including violence, assault, and robbery. With the advent of widely accessible information and communication technologies (ICTs), sex workers have begun to use electronic occupational health and safety tools to mitigate these risks.

**Objective:**

This study aims to explore the use of ICTs by sex workers for managing occupational health and safety risks and strategies for reducing these risks. This paper aims to answer the following question: what is known about sex workers’ use of ICTs in the delivery of occupational health and safety strategies?

**Methods:**

A literature review following the methodological framework for scoping reviews was conducted to analyze studies describing the use of ICTs by sex workers to mitigate occupational health and safety risks. Experimental, observational, and descriptive studies, as well as protocol papers, were included in this scoping review.

**Results:**

Of the 2477 articles initially identified, 41 (1.66%) met the inclusion criteria. Of these studies, 71% (29/41) were published between 2015 and 2019. In these studies, the internet was the predominant ICT (24/41, 58%), followed by text messaging (10/41, 24%) and assorted communication technologies associated with mobile phones without internet access (7/41, 17%; eg, voice mail). In 56% (23/41) of the studies, sex workers located in high-income countries created occupational health and safety strategies (eg, bad date lists) and shared them through the internet. In 24% (10/41) of the studies, mostly in low- and middle-income countries, organizations external to sex work developed and sent (through text messages) occupational health and safety strategies focused on HIV. In 20% (8/41) of the studies, external organizations collaborated with the sex worker community in the development of occupational health and safety strategies communicated through ICTs; through this collaboration, concerns other than HIV (eg, mental health) emerged.

**Conclusions:**

Although there has been an increase in the number of studies on the use of ICTs by sex workers for managing occupational health and safety over the past 5 years, knowledge of how to optimally leverage ICTs for this purpose remains scarce. Recommendations for expanding the use of ICTs by sex workers for occupational health and safety include external organizations collaborating with sex workers in the design of ICT interventions to mitigate occupational health and safety risks; to examine whether ICTs used in low- and middle-income countries would have applications in high-income countries as a substitute to the internet for sharing occupational health and safety strategies; and to explore the creation of innovative, secure, web-based communities that use existing or alternative digital technologies that could be used by sex workers to manage their occupational health and safety.

## Introduction

### Background

Sex workers encounter a number of occupational health and safety risks. In Germany [1], Thailand [2], Ireland [3], Israel [4], Cambodia [5], and Canada [6-8], studies have reported that sex workers are subjected to violence, assault, and harassment. These risks are experienced by transgender individuals who identify as women [6], cisgender women [7-9], cisgender men [10], and transgender sex workers who identify as men [10]. Although there are many categories of sex work, this paper will focus on sex work where a sex worker and their client have physical contact as commercial sex work [11].

To counteract these occupational health and safety risks, sex workers have begun to use information and communication technologies (ICTs)—most commonly, the internet and social media—to exchange tips and information [12]. In a study composed of a literature review and interviews with sex workers that was published in 2016, the Global Network of Sex Work Projects (NSWP) reported that ICTs are a new type of business tool for sex workers that has enabled a global change from outdoor sex work to indoor sex work [12]. In addition, their study reported that sex workers in high-income countries use the internet to advertise their services. In low- and middle-income countries (LMICs), sex workers provide regular clients with their phone numbers and receive calls from potential clients. In consultation with sex workers in 7 countries, ranging from low- to high-income countries, the NSWP reported that ICTs provide sex workers with the means to screen clients as well as set the terms of the encounter in advance of appointments with clients. This has led to a decrease in the rate of violence perpetrated against sex workers [12]. The predominant method for accessing ICTs was found to be through a smartphone [12]. The same study posited that the use of ICTs enhanced social cohesion among sex workers [12], which can lead to beneficial effects among sex workers in reducing occupational health and safety risks [13,14]. In addition, the NSWP highlighted in their review that the World Health Organization has published data on the value of ICTs in promoting condom use [12].

### Objectives

This scoping review aims to answer the following research question: what is known about sex workers’ usage of ICTs in the delivery of occupational health and safety strategies? Specifically, this study seeks to examine the types of ICTs used by sex workers, the types of occupational health and safety strategies delivered through ICTs, and the individuals and organizations engaged in the development of occupational health and safety strategies. A thorough search of the literature did not reveal any systematic reviews or scoping reviews on this topic.

## Methods

### Literature Review Strategy

A literature review consistent with the Preferred Reporting Items for Systematic Reviews and Meta-analyses Extension for Scoping Reviews guidelines and following the methodology pioneered by Arksey and O’Malley [15] and enhanced by Levac et al [16] was used to analyze studies on sex workers’ use of ICTs in the delivery of occupational health and safety strategies.

### Inclusion Criteria

Peer-reviewed studies were included if they met the following criteria: (1) published in English and (2) described an intervention in which sex workers used ICTs for occupational health and safety strategies (occupational health and safety strategies include, but are not limited to, violence prevention, having a buddy system, managing the effects of stigma on mental health, screening clients, condom use when providing sexual services, and clearly stating boundaries during the encounter), ICTs were developed for sex workers to apprise them of occupational health and safety strategies, and ICTs were developed with sex workers to exchange and communicate occupational health and safety strategies. Experimental, observational, and descriptive studies analyzing website contents, as well as protocol papers, were screened and included for review if the inclusion criteria were met. No limitations with regard to the year of publication were imposed.

### Search Strategy

A comprehensive literature search was conducted in September 2019 using the following databases: MEDLINE, Embase, PsycINFO, CINAHL, and ProQuest Dissertations and Theses.

Search strategies were developed with the assistance of a professional librarian (Erica Lenton). Key concepts for the database search included sex worker and ICTs. The concept of occupational health and safety was screened manually because including it as a concept in the database search limited the results and excluded relevant papers. The final search strategy for MEDLINE can be found in Multimedia Appendix 1.

### Selection Procedure

**Figure 1:**
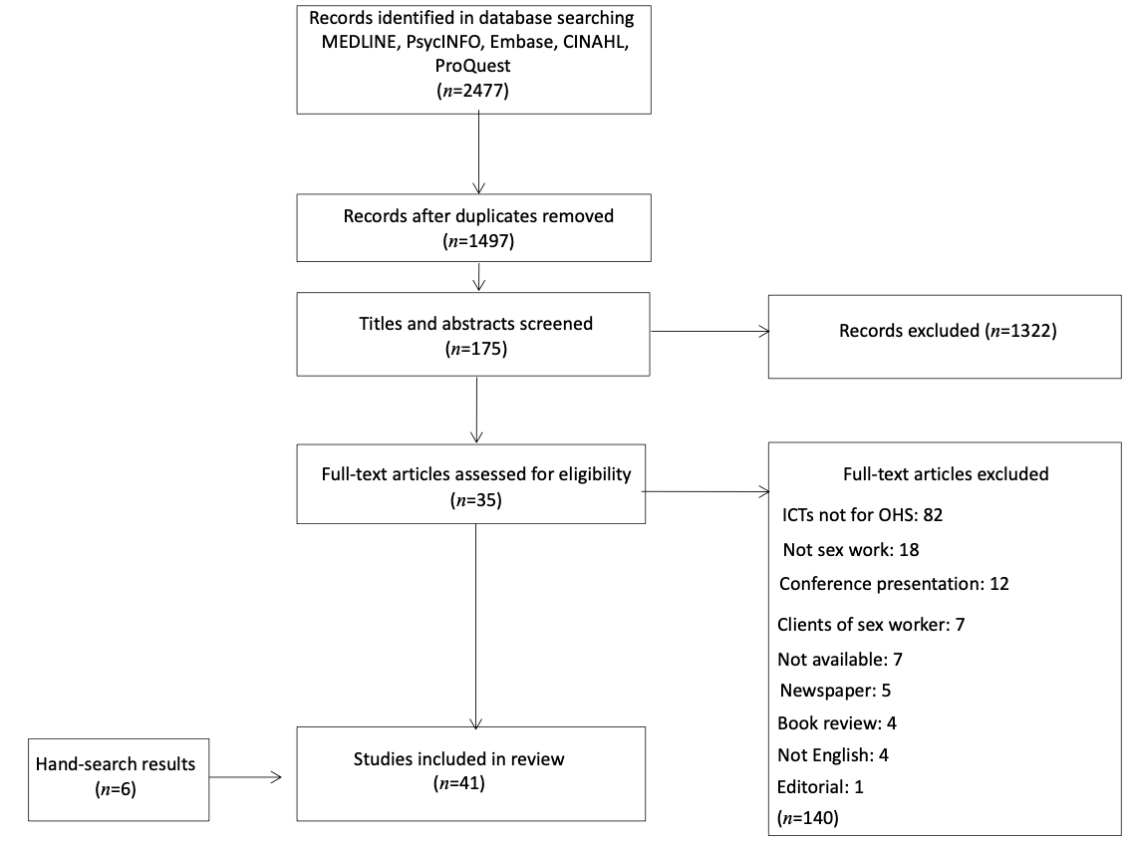
Preferred Reporting Items for Systematic Reviews and Meta-Analyses flowchart. ICT: information and communication technology; OHS: occupational health and safety.

The selection criteria forms were developed by the primary author (TB). The initial database search resulted in 2477 articles returned (629 from MEDLINE, 713 from Embase, 420 from PsycINFO, 282 from CINAHL, and 433 from ProQuest Dissertations and Theses). The selection procedure is illustrated in the flow diagram in Figure 1. After removing 980 duplicates, 1497 articles remained. By applying the inclusion criteria, title and abstract screening was performed independently by TB and AS, resulting in 1322 excluded articles, leaving 175 articles for full-text review. The full-text review was performed independently by TB and AS. After the full-text review, 35 articles were selected for inclusion in the scoping review, and 140 were excluded. These 140 articles were excluded for the following reasons: (1) ICTs were not used for occupational health and safety strategies; (2) the article was not about sex work; (3) the article was a conference presentation (poster, oral, and plenary); (4) the article only addressed clients of sex workers and not sex workers; (5) the article was not available (eg, no longer in print); (6) the article was a newspaper article or book review; (7) the article was written in a language other than English; and (8) the article was an editorial. Disagreement between the reviewers TB and AS was resolved by discussion until a consensus was reached. A secondary search was performed by TB, consisting of screening the reference list of the included articles, which yielded 5 articles. A query to a sex work research network (Sex Work Research Hub) netted an additional single article. The search resulted in a total of 41 articles included for full data extraction and analysis. The final search strategy can be found in Multimedia Appendix 1.

### Data Extraction

A data charting table was developed by TB, discussed with AS, and consensus was reached on the table elements that included (1) research design and if sex workers were included in the design, (2) research ethics board approval, (3) aim and goals of study, (4) type of sex work and location, (5) legislative models, (6) specific study population, (7) characteristics of the study population, (8) type of ICT, (9) occupational health and safety addressed, (10) source of occupational health and safety strategy, (11) occupational health and safety strategy, and (12) main findings. These elements were chosen to reflect the types of sex work and the associated occupational health and safety risks. TB and AS each independently charted 5 articles and then discussed the results to ensure consistency in their understanding of each table element. TB and AS then proceeded to independently complete the data extraction for all 41 articles. Any disagreement between TB and AS was resolved through discussion until a consensus was reached. At the completion of data extraction, themes were identified by examining the exposure that sex workers face with regard to occupational health and safety risks, the provenance of occupational health and safety strategies, and the ICTs used to communicate these strategies.

## Results

### Study Characteristics

Of the 41 studies selected for review, 7 were experimental (5 were randomized controlled trials) [17-23], 19 were observational studies [10,24-41], 13 were descriptive studies analyzing website contents [42-54], and 2 were protocol papers [55,56]. Each paper was a unique separate study, meaning that no two papers discussed the same data.

A total of 5 studies were conducted in high-income countries (Australia, Canada, China, the United Kingdom, and the United States), and 7 studies were conducted in LMICs (Cambodia, India, Kenya, Mexico, Mozambique, South Africa, and Zimbabwe).

Study publication dates ranged from 2004 to 2019, with 71% (29/41) of the studies published between 2015 and 2019. Since 2007, according to a study published by the NSWP in 2017, ICT use by sex workers, particularly the internet and smartphones, has risen, reflecting the pace of growth in the general population [12]. For example, 33% of the Canadian adults in 2012 were using smartphones. In 2013, this percentage increased to 56% [57]. By 2018, 90% of the Canadian adults were using smartphones [58].

### Main Themes

The main themes identified in the literature were related to (1) sex work context, (2) ICTs, (3) sex workers’ implementation of occupational health and safety strategies through ICTs, (4) implementation of occupational health and safety strategies through ICTs by organizations external to sex workers, and (5) researchers collaborating with sex workers in the study of ICTs used for occupational health and safety strategies. Representative studies (exemplars) are presented and discussed for themes 3, 4, and 5.

### Sex Work Context

Within the sex work context, four concepts were identified: occupational health and safety concerns, gender, sex work venue, and legislative models.

The 41 papers reported on one or more of the following categories of occupational health and safety concerns: (1) sexually transmitted infection and HIV prevention, testing, and risk behaviors (13 studies); (2) risk management practices (13 studies); (3) sexual and reproductive health (3 studies); (4) hepatitis B virus prevention (1 study); (5) drug use (1 study); and (6) stigma (1 study). The occupational health and safety concerns and associated studies are presented in Table 1.

Of the included studies, 41% (17/41) pertained to cisgender women [18,19,21,26-30,34,36,37,39,46,50,55,56,59]. Approximately 27% (11/41) were on cisgender men [20,31,32,40,42,43,48,51-54], and 7% (3/41) addressed the following genders: cisgender women, cisgender men, and transgender women [33,44,49]. Of the studies, 4% (2/41) addressed cisgender women, cisgender men, and transgender individuals whose gender identity was not specified [41,45], and 12% (5/41) of the studies did not specify a gender [17,22,23,25,35]. Approximately 5% (2/41) of the studies pertained to cisgender women and cisgender men [24,47], and 1 study addressed cisgender men and transgender sex workers who identified as men [10]. No studies were found that focused solely on transgender sex workers.

The sex work venue is generally regarded as indoor, outdoor, or both. Of the 13 studies that did not specify the venue, extrapolation was performed to determine the venue. Extrapolation was based on studies taking place in the same country or used information contained in the study or used other reliable sources (such as government websites). A total of 14 studies specifically mentioned indoor sex work as the type of venue in which sex workers conducted their business [25,26,31,33,34,37,43,47-52,56]. A total of 6 additional studies were extrapolated to be indoor [22-24,42,53,54]. Venues that were specified as “internet-based” [45] or “web-based” [44] were considered to be indoor sex work. Sex tourism was categorized as indoor based on the content of the study [46]. A total of 3 studies mentioned outdoor (street-based) sex work [9,17,40]; 2 were extrapolated to be outdoors [18,55]; and 8 studies addressed occupational health and safety for both indoor and outdoor (street-based) sex workers [10,27-30,35,36,59], with 5 studies extrapolated to be as such [20,21,32,39,41]. Of the 41 studies, 23 (56%) addressed indoor sex work, 5 (12%) addressed outdoor sex work, and 13 (32%) addressed sex workers in both indoor and outdoor settings.

Legislative models pertaining to sex work for each country in the study are shown in Table 2. Legislative models are categorized as (1) full criminalization, (2) partial criminalization, (3) criminalization of purchase of sex, (4) regulatory models, and (5) full decriminalization (definitions included in the notes for Table 2) [60]. Sex work is deemed to be a criminal activity in 83% (10/12) of the countries in the papers selected for this study and is regulated in 17% (2/12) of the countries in the study.

**Table 1:** Occupational health and safety concerns for indoor, outdoor, and indoor and outdoor sex workers.

OHS^a^ addressed			Gender								
			Cisgender woman		Cisgender man		Transgender individual		All genders		Not specified
**STI^b^ and HIV prevention and testing**											
	STI prevention [25]	I&O^c^		—^d^		—		—		I	
	STI testing [17,36,56]	I^e^, I&O		—		—		—		—	
	STI: Syphilis [51]	—		I		—		—		—	
	Regular HIV testing [17,36]	I&O		—		—		—		O^f^	
	HIV self-testing [18,19,56]	O, I		—		—		—		—	
	Reattend for HIV and STI testing [23]	—		—		—		—		I	
	HIV prevention [20,26,27,32,37,40,41]	I, I&O		I&O, O		—		I		—	
	Reduction of HIV and STI incidence [21]	I&O		—		—		—		—	
	ART^g^ adherence [39]	I&O		—		—		—		—	
	Safe sex practices with clients [17,25,31,52,56]	I, I&O		I		—		—		I, O	
	Condomless sex [20,46,49,50,54]	I		I, I&O		I (women)		I		—	
	Inconsistent condom use [26,28-30]	I, I&O		—		—		—		—	
**Risk management practices**											
	Risk management [34,42,44,47]	I		I		I (women)		—		—	
	Personal safety [43,45]	—		I		—		I		—	
	Violence [10,33,59]	I, I&O		I		I (identify as men)		I		—	
	Violence prevention [35]	—		—		—		I&O		—	
	Virtual violence [34]	I		—		—		—		—	
	Electronic abuse [34]	I		—		—		—		—	
	Verbal abuse [34]	I		—		—		—		—	
	Harassment [59]	I&O		—		—		—		—	
	Harassment through verbal abuse, repeated or unwanted contact or attempts to contact through email, text, or social media [33]	—		—		—		I		—	
	Stalking through verbal abuse, repeated or unwanted contact or attempts to contact through email, text, or social media [33]	—		—		—		I		—	
	Murder [33]	—		—		—		I		—	
	Nonpayment [33]	—		—		—		I		—	
	Attempts to underpay [33]	—		—		—		I		—	
**Sexual and reproductive health**											
	Sexual and reproductive health [24,47,55,56]	O, I		I		—		—		—	
	Health status [46,48,53]	I		I		—		—		—	
	Sex worker health [45]	—		—		—		I			
HBV^h^ prevention [22]			—		—		—		—		I
Drug use [51]			—		I		—		—		—
Stigma [10]			—		I&O		I&O (identify as men)		—		—

^a^OHS: occupational health and safety.

^b^STI: sexually transmitted infection.

^c^I&O: indoor and outdoor.

^d^Not available. Either no studies were found for a specific gender, or the studies found addressed all genders, or the studies did not specify a gender.

^e^I: indoor.

^f^O: outdoor.

^g^ART: antiretroviral therapy.

^h^HBV: hepatitis B virus.

**Table 2:** Legislative models^a,b^.

Country^c^	Articles found, n (%)^d^	Full criminalization	Partial criminalization	Criminalization of purchase of sex	Regulatory models	Full decriminalization
South Africa [17]^e^	1 (2)	✓				
Zimbabwe [17]^e^	N/A^f^		✓ [61]			
Mozambique [17]^e^	N/A		✓ [62]			
Kenya [18,19,55]	3 (7)	✓ [61]				
India [20,28-30,36,39,40,59]	8 (21)		✓			
Cambodia [37,56]	2 (5)	✓ [63]				
China [26,27]	2 (5)	✓ [64]				
Australia [22,24,42,43,49]	5 (12)				✓	
United Kingdom [23,33,34,47,50]	5 (12)		✓			
United Kingdom, Canada, and Australia [32]	1 (2)		✓ (United Kingdom)	✓ (Canada)	✓ (Australia)	
Canada [10,35,45]	3 (7)			✓		
United States [44,51-54]	5 (12)	✓				
United States and Canada [25]	1 (2)	✓ (United States)		✓ (Canada)		
United States and United Kingdom [48]	1 (2)	✓ (United States)	✓ (United Kingdom)			
Mexico [21]	1 (2)				✓	
Global [41,46]	2 (5)					
Unknown (country not specified) [31]	1 (2)					
Total	41 (100)	5 countries	4 countries	1 country	2 countries	N/A

^a^The definition of each legislative model was obtained from Platt et al [60]. *Full criminalization:* all aspects of selling and buying sex or organization of sex work are prohibited. *Partial criminalization:* organization of sex work is prohibited, including working with others, running a brothel, involvement of a third party, or soliciting. *Criminalization of purchase of sex:* often referred to as the sex-buyer model—laws penalize sex workers working together (under third-party laws), any aspect of participating in the sex trade as a third party, and buying sex. *Regulatory models:* the sale of sex is legal in licensed models or managed zones and is often accompanied by mandatory condom use, HIV and sexually transmitted infection testing, or registration. *Full decriminalization:* all aspects of adult sex work are decriminalized, but condom use is legally required in some locations (eg, New Zealand).

^b^Unless otherwise indicated, data on legislative models were obtained from Platt et al [60].

^c^A total of 12 countries in study.

^d^Percentages have been rounded.

^e^South Africa, Zimbabwe, and Mozambique were documented in the same study.

^f^N/A: not applicable.

### Use of ICTs

The internet, including websites and web-based activities (defined as performing activities such as participating in a discussion forum, researching a potential client’s credentials, using email, or being active on social media), accounted for 58% (24/41) of the studies. The remainder of the studies investigated the use of mobile phones without internet access; of these, 24% (10/41) examined text messaging, and the other 17% (7/41) looked at assorted communication technologies associated with mobile phones without internet access, such as phone calls, interactive voice response (IVR), audio broadcast messaging, and voice mail.

### Sex Workers’ Implementation of Occupational Health and Safety Strategies Through ICTs

In 56% (23/41) of the studies, occupational health and safety strategies were initiated by the sex workers themselves, using the internet (through computers or smartphones), text messaging, or mobile phones without internet access.

Using the internet, indoor sex workers were found to use an array of electronic strategies to implement risk management practices for screening clients. In the United Kingdom and Montréal (Canada), screening tools such as electronic bad-date lists (a bad-date list is a list consisting of descriptions of clients that have been abusive (eg, violence) toward sex workers) were made available to sex workers through sex worker–run organizations [33,35]. Indoor sex workers also searched for information on potential clients by consulting closed sex worker Facebook groups and web-based sex worker forums and by using Google (Alphabet Inc). Prebooking clients and negotiating services and fees through email or web chats were all part of the occupational health and safety strategies used by sex workers to screen potential clients. Through these strategies, sex workers can assess a potential client for violence, nonpayment, and other abusive behavior. These strategies were used by indoor sex workers in Vancouver (Canada) [10], the United Kingdom [33,34], and the United States [44]. Sex workers may also report abusive content to their website administrator because anonymous web-based communication can sometimes result in threats [34] or harassment [33]. In addition, by using the internet, indoor sex workers communicated safe work practices on their website advertisements. In Vancouver (Canada), sex workers advertised the use of condoms and emphasized no drugs and no alcohol during the encounter; cisgender women specified in their advertisements the services that would be provided during client encounters [45]. Sex workers in Detroit (United States) using a web-based service to advertise their services specified the services they would provide during client encounters [44]. Similar practices were used by sex workers in the United Kingdom, Florida (United States), and Sydney (Australia) through communications through web-based advertisements [47,49,52]. With the two-way electronic communication capabilities offered by the internet and with access to the internet through mobile phones, sex workers managed the risks inherent in their profession by engaging in a dialog with clients regarding their acceptable business practices, also known as safe work practices, which mitigated their occupational health and safety risks.

As another example of sex worker–initiated occupational health and safety strategies, sex workers in the United Kingdom used the text messaging app WhatsApp (Facebook, Inc) to form private chat groups to exchange information on clients [33]. In addition, in the United Kingdom, sex workers have implemented an occupational health and safety strategy known as the “buddy system”; this strategy consists of a sex worker using a mobile phone to text a colleague to apprise them about scheduled client encounters, the location of the encounters, and start and end times of the encounters [34].

Verbally communicating with clients using mobile phones is yet another sex worker–initiated occupational health and safety strategy. In India, mobile phones without internet access are the most frequently used ICT. The main occupational health and safety strategy of cisgender women was negotiating condom use with regular clients and potential clients through phone calls [28,30]. The possibility of successfully convincing the client to use a condom was dependent on the cisgender woman’s income streams. If she had no other income apart from sex work, the odds of providing services without a condom were higher [28,30].

### Implementation of Occupational Health and Safety Strategies Through ICTs by Organizations External to Sex Workers

In 44% (18/41) of the studies, occupational health and safety strategies were found to be created by organizations external to sex work. These organizations were composed of researchers, public health agencies (PHAs), community-based organizations, and social enterprises working either separately or together. In the studies, the predominant occupational health and safety concern of these organizations was the transmission and prevention of sexually transmitted infection and HIV. The strategies created by these external organizations were disseminated by using the internet, text messaging, and audio broadcast messages through mobile phones. In 24% (10/41) of the studies, occupational health and safety strategies were developed without an explicit indication of sex workers’ input. The next 4 exemplar studies highlight the internet and text messaging as the ICTs used.

Using the internet, and without describing whether collaboration with sex workers occurred on occupational health and safety strategy content, external organizations have reached out to sex workers to apprise them of sexually transmitted infection and HIV prevention strategies. In the first exemplar, in the 2000s (early 21st century) in the United States, PHAs and community-based organizations initiated a health information dissemination campaign by engaging with cisgender male sex workers and their clients in web-based forums and chat rooms in 8 US cities to discuss the risks and prevention of syphilis and HIV [51]. In this study, the forum participants in New York City engaged the PHAs in discussions regarding the risks of condomless sex [51]. The next two exemplars are from China; collaboration with sex workers was not specified in either study. Two quantitative studies examined the feasibility of using the internet to communicate HIV prevention information to indoor cisgender female sex workers [26,27]. In the first study, web-based public service announcements were used to attempt to reduce inconsistent condom use among indoor cisgender female sex workers, with a small percentage of sex workers (6.7%) reporting that they were using condoms consistently after seeing the announcements [26]. In the second study, most of the sex workers surveyed (64%) indicated that they would join a web-based HIV and sexually transmitted infection prevention program [27].

The fourth exemplar features text messaging. In this study, HIV testing was the focus for outdoor cisgender female sex workers in sub-Saharan African countries. The North Star Alliance, a social enterprise, operates roadside wellness clinics in Africa. It designed and implemented a text messaging intervention for HIV prevention by formulating the content of text messages without sex worker input. A cluster randomized controlled trial with two arms was conducted, with one arm receiving the text messaging intervention (the other arm received verbal HIV prevention information at the clinic). A total of 167 sex workers were part of the intervention arm; they were located in South Africa, Zimbabwe, and Mozambique. The text messages encouraged the study participants to use condoms during sex and obtain regular HIV testing. A total of 35 messages were sent over a 29-week period. The study found that condomless sex did not diminish in the cohort using text messaging, but there was an increase in self-reported HIV testing [17].

In 20% (8/41) of the studies, organizations external to sex workers collaborated with the sex worker community in the development of occupational health and safety strategies, as demonstrated in the following four exemplars that highlight these ICTs: IVR, audio broadcast messaging, text messaging, and voice mail messaging.

In the first exemplar, a pilot study in Kolkata, India, was conducted to examine the potential of daily messaging using IVR to improve antiretroviral therapy adherence in people living with HIV. The IVR messages were developed with a community advisory board composed in part of sex workers who were part of a peer support network for sex workers. Brief messages (less than 1 min in duration) focused on strategies for self-management of three domains: medical, mental health, and nutrition and hygiene. In addition, 3 antiretroviral therapy appointment reminders were sent each month. A total of 46 participants were enrolled in the study, and 65% (30) were cisgender female sex workers. After the 1-month pilot, focus groups were convened with the participants to provide feedback on the IVR messages. The results of the focus group discussions demonstrated appreciation for the health and wellness messages. The results after 1 month indicated that medication adherence improved [39].

In the second exemplar, in an ethnographic study in Bangalore, India, audio broadcast messages were formulated by outreach workers with lived urban sex worker experience. The goal of these broadcast messages was to remind urban sex workers (cisgender women) to get tested for HIV and syphilis. In the study, the messages were sent to 230 phone numbers; 121 recipients listened to the entire message [36].

The third exemplar is a study that was conducted in Mexico at 2 locations: Tijuana, with 141 cisgender women, and Ciudad Juarez, with 129 cisgender women. Focus groups with 25 cisgender women in Tijuana provided feedback on the content and relevance of the text messages. There were two types of text messages. One type was short-term oriented, for example, encouraging cisgender women to have condoms handy before they went out for the evening. The second type was more long-term (future) oriented; for example, although condoms are expensive, the gains achieved in maintaining one’s health are worth the investment. The text messages were randomly assigned to participants in both cities in the study. The goal of the study was to evaluate the impact of the short- and long-term text messages that were designed to maintain improvements in safer sex practices among drug-using and nondrug-using cisgender female sex workers. The outcome measure was the HIV and sexually transmitted infection incidence rate over the 6-month follow-up period. Cisgender women in Tijuana, where there was a lower percentage of hard drug use, benefited from future-oriented text messages that focused on the advantages of long-term safer sex maintenance practices to achieve future-oriented goals. In contrast, cisgender women in Ciudad Juarez, where there was a higher percentage of methamphetamine, cocaine, heroin (ie, hard drugs), and injectable drug use, did not benefit from either the short-term or future-oriented text messages [21].

The fourth exemplar is a pilot study conducted in Chennai, India. The initial goal of the pilot was to assess whether HIV prevention messages sent by either voice mail or text messages would be acceptable to cisgender male sex workers. Message construction was based on the results of the interviews and focus groups that were conducted using semistructured questions. In addition to acceptable message content for the HIV prevention intervention, the researchers asked the study participants if there were future additional services that they would like to receive. The cisgender men voiced their concerns regarding sexual health, negotiating condom use with clients, stigma, mental health, tuberculosis prevention, and assistance in exiting sex work [40].

The results found in the fourth exemplar were also observed in a number of studies included in this scoping review, meaning that when organizations external to sex work applied participatory methods to the research design, other issues, in addition to the prevention and transmission of sexually transmitted infection and HIV [20,21,36,39,40,56], surfaced. Specifically, sex workers voiced their concerns regarding sexual and reproductive health [40,55], stigma [40], mental health [39], tuberculosis prevention [40], and assistance in exiting sex work [40].

### Researchers Surveying Sex Workers’ Use of ICTs for Occupational Health and Safety Strategies

Although external organizations worked collaboratively with sex workers to develop occupational health and safety strategies, as illustrated in the aforementioned four exemplars, the use of collaborative methods such as participatory action research (PAR) and community-based participatory research (CBPR; PAR and CBPR come under the umbrella of participatory methods. Participatory methods in research emphasize research with, not on, communities and are a collaborative form of research involving the community in all aspects of the research [65,66]) were applied to studies that involved a collaboration between the sex worker community and researchers endeavoring to investigate the use of ICTs by sex workers for managing occupational health and safety. In the included studies for this scoping review, three collaborations took place in 2 high-income countries, the United Kingdom [33] and Canada [10,35], and one in India, an LMIC [59].

The next two exemplars demonstrate these collaborations. In Vancouver (Canada), a collaboration between researchers and members of the sex work community analyzed the content of qualitative interviews conducted with indoor and outdoor cisgender men and transgender individuals who identify as men to explore the safety aspects involved in transitioning from outdoor to internet-based indoor sex work, such as using webchats to assess a potential client’s propensity for violence [10]. In moving to indoor sex work facilitated through the internet, the men in Vancouver (Canada) experienced less stigma perceived to be because of reduced visibility on the street [10]. However, the study found that not all sex workers in the community shifted to an indoor work environment. With more clients having followed the shift to having services provided indoors, fewer clients were available to the street-based sex workers, with an associated loss of income for these sex workers. The study found that the sex workers made up the loss of income by offering services such as anal sex that could be detrimental to their health [10].

The other exemplar was conducted in India [59]. A collaborative effort with sex workers, nongovernmental organizations, and academics resulted in the development of interview guides to study the challenges of using mobile phone communication for indoor and outdoor cisgender female sex workers. Using a mobile phone without internet access to solicit clients assisted the sex workers in moving their work indoors and avoiding the harassment associated with outdoor work [59].

In both studies, the sex workers expressed that the move to indoor work resulted in a decrease in violence, stigma, and harassment. However, the sex workers in both countries experienced a sense of loss of community because they no longer saw each other on the strolls (ie, on the streets) [10,59].

## Discussion

### Principal Findings

This scoping review examined the occupational health and safety risks experienced by sex workers and the use of ICTs as an occupational health and safety tool to mitigate these risks. Of the 41 studies included, 29 were published between 2015 and 2019, indicating that sex workers have begun to use a number of electronic tools to mitigate the occupational health and safety risks present in their profession. The included studies revealed that sex workers used one or more of the following ICTs: (1) the internet, (2) mobile phones, (3) messaging technology through mobile phones (IVR, voice mail, text, and audio broadcast messaging), and (4) social media apps. The findings suggest that the type of ICT used by sex workers is shaped by the country of employment. In high-income countries, the internet has become a commonly accepted commercial sex venue [67]. Moreover, internet use in high-income countries is greater than that in LMICs [68] and social media apps are commonly used. In LMICs, the predominant ICT is text messaging through mobile phones.

In 83% (10/12) of the countries included in this review, sex work is criminalized, and in 17% (2/12), it is regulated. No studies were found that addressed sex work in a country where it was decriminalized (in New Zealand, where decriminalization has occurred, sex workers are protected by labor laws that enhance their occupational health and safety, and mobile phones are considered an essential safety tool [69]). Legislative models that criminalize sex work in the country of employment were just one of the structural determinants that influenced a sex worker’s occupational health and safety risks [70]. Other structural determinants such as working conditions, location, and income outside of sex work [28,30,71] all affected whether a condom was used during the provision of services and the type of services provided [10,50]. In public health studies, condom use is the recommended method for preventing the acquisition and transmission of sexually transmitted infections and HIV, and regular and frequent testing is also recommended to detect possible acquisition. In LMICs, the occupational health and safety strategies delivered through ICTs found in the included studies reflected this public health approach. However, these studies did not address possible financial and societal barriers that could impede sex workers—particularly women—from accessing HIV testing [72].

The criminalization of sex work and the accompanying stigma, as well as the technology available in high-income countries and LMICs, directed the choice of ICTs used by sex workers. In collaborative research projects in LMICs, discretion in receiving occupational health and safety risk mitigation strategies was the overriding concern; therefore, IVR, voice mail, and audio broadcast messaging were the preferred communication methods, as opposed to a text message that could inadvertently be seen by a family member on a sex worker’s mobile phone [36,39,40]. In high-income countries, the internet is the prevalent ICT. However, the use of the internet has been impeded by the passage of the 2018 Allow States and Victims to Fight Online Sex Trafficking Act (FOSTA) and Stop Enabling Sex Traffickers Act (SESTA) in the United States, which prohibit sex work content on websites, including harm reduction techniques and advertising [73-75]. Backpage [76] was a popular site for sex worker advertisements [44], but after the passage of the acts, its management no longer allowed sex workers to advertise on the site. The FOSTA-SESTA have also affected Canadian sex workers because 60% of the sex workers used to advertise on Backpage [76] before it was banned [77]. Craigslist, a popular web-based classified advertisements site in Toronto (Canada) and environs, has also banned all sex work–related advertisements [78]. Sex worker organizations are concerned that the safety precautions that internet-based sex workers were able to take are now disappearing because sex workers are being pushed back to outdoor work [73,77]. A recommended area of research would be to examine whether the use of text messaging, IVR, voice mail, and audio broadcast messaging could serve as safe alternative electronic spaces in high-income countries to replace those that have disappeared, not only for advertising purposes but also for sex workers to be able to once more create secure online communities to manage the structural determinants that constrain their health and safety. However, the protection of information communicated through these ICTs would depend on the laws governing electronic communication in these countries (in Canada, the Supreme Court has ruled that electronic conversations are protected by that country’s privacy laws [79]). A further recommendation is to examine the creation of new secure online communities using existing or alternative digital technologies, for example, Red Cloud [80] in Australia, a web hosting enterprise that does not come under the FOSTA-SESTA.

This review found that the predominant occupational health and safety concern of organizations external to sex work was the transmission and prevention of sexually transmitted infection and HIV, and the ICT interventions developed by these organizations consisted of pushing sexually transmitted infection and HIV prevention and testing messaging out to either the internet or to sex workers’ mobile phones. Only a small number of studies included in this scoping review used collaborative research methods such as PAR and CBPR to either design or study an ICT intervention. In these collaborative studies, sexual and reproductive health, stigma and its associated mental health effects, and violence emerged as the occupational health and safety risks that sex workers were most concerned about [10,33,40,56,59]. PAR and CBPR fall under the umbrella of participatory methods. When sex workers are involved in the research project as members of the research team, the research is no longer a passive exercise [81]. The sex workers’ agency is leveraged, and their experiences, both personal and professional, are discussed. Through participatory research methodology used in previous studies with sex workers, structural determinants that affect sex workers’ occupational health and safety, such as violence [82], stigma, social norms, lack of support from family and friends [82,83], and social support from community resources and sex worker colleagues emerged, as well as their resilience skills in managing their circumstances [83]. On the basis of these findings, it is recommended that future research involve sex workers in the formulation of the research question and scope of the study to accurately identify the issues that sex workers face in their personal and professional lives. A further area of research would be to compare the longevity and continued effectiveness of interventions designed with sex workers with those designed without sex worker input.

### Limitations and Strengths

A limitation of this review is that, for feasibility reasons, only studies published in English were included. By limiting the studies to English, relevant findings may have been missed. In addition, the gray literature was not consulted, which may have resulted in novel ways of using ICTs by sex workers not being represented. Another limitation is that no studies were found that focused solely on transgender sex workers, and studies that did include transgender individuals combined them with cisgender women and cisgender men.

The strengths of this review include the rigor of the methods, including the use of duplication at each phase of the review (article screening and selection, data extraction, and full-text analysis), the absence of limitations with respect to year of publication, and the inclusion of a wide range of study designs. In addition, this study provides a broad overview of the different aspects of ICT use among sex workers across the globe because studies published during the last 15 years were included to account for recent developments in electronic communications technology.

### Conclusions

This study provides important insights into how the use of ICTs during the first two decades of the 21st century has shaped the methods that sex workers use to keep themselves safe on the job in environments where sex work is criminalized*.* This review found that a considerable amount of research on ICT use demonstrated that internet-based sex workers, that is, sex workers using the internet to conduct business, were active creators and sharers of the occupational health and safety strategies used in their work. This review also found that researchers, PHAs, and social enterprises use text messaging as their ICT of choice for pushing sexually transmitted infection and HIV prevention and testing messages out to sex workers’ mobile phones, although this is not the main occupational health and safety concern for sex workers. Only a small number of studies in this review engaged sex workers in collaborative research; yet, these collaborations yielded insights into the lives of sex workers and provided direction for future research efforts. Applying participatory methods that engage sex workers of all genders as active participants in the research process will produce applications of digital technology geared toward their occupational health and safety needs.

## Appendix

Multimedia Appendix 1 MEDLINE search strategy.

## Abbreviations

CBPR: community-based participatory research
FOSTA: Fight Online Sex Trafficking Act
ICT: information and communication technology
IVR: interactive voice response
LMIC: low- and middle-income country
NSWP: Global Network of Sex Work Projects
PAR: participatory action research
PHA: public health agency
SESTA: Stop Enabling Sex Traffickers Act
