# Supplementary material for: The Use of Information and Communication Technologies by Sex Workers to Manage Occupational Health and Safety: Scoping Review
Source: J Med Internet Res. 2021 Jun 24;23(6):e26085. doi: 10.2196/26085 (PMC8277340; doi:10.2196/26085)
Supplement: Multimedia Appendix 1 [file jmir_v23i6e26085_app1.docx]

Multimedia Appendix 1 – Search strategy. Medical Literature Analysis and Retrieval System Online search strategy conducted on September 23, 2019

Subject headings, exploded subject headings and keywords applied to these two concepts: sex worker, and information and communication technologies (ICTs), made up the search strategy.

1. ((exot* or erot*) adj3 danc*).tw,kf.

2. girlfriend experience.tw,kf.

3. sex worker.mp. or exp Sex Workers/

4. (mal* adj1 prostitut*).tw,kf.

5. (mal* adj1 sex work*).tw,kf.

6. madam.tw,kf.

7. mammy.tw,kf.

8. (indoor adj1 sex adj1 work*).tw,kf.

9. (outdoor adj1 sex adj1 work*).tw,kf.

10. (migrant adj1 sex adj1 work*).tw,kf.

11. (trans* adj1 sex adj1 work*).tw,kf.

12. (transaction* adj1 sex adj1 work*).tw,kf.

13. streetwalk*.tw,kf.

14. money boy.tw,kf.

15. (female adj1 entertainment adj1 work*).tw,kf.

16. escort.tw,kf.

17. prostitut*.tw,kf.

18. brothel.tw,kf.

19. rent boy.tw,kf.

20. hustler.tw,kf.

21. exp Sex Work/

22. (sex adj1 work*).tw,kf.

23. video performer.tw,kf.

24. webcamming.tw,kf.

25. (marg* adj1 sex work*).tw,kf.

26. (men adj1 who adj1 purchase adj1 sex).tw,kf.

27. (sex adj1 purchasing).tw,kf.

28. (indirect adj1 sex adj1 work*).tw,kf.

29. (direct adj1 sex adj1 work*).tw,kf.

30. (indirect adj1 female adj1 sex adj1 work*).tw,kf.

31. (direct adj1 female adj1 sex adj1 work*).tw,kf.

32. brothel-based.tw,kf.

33. street-based.tw,kf.

34. lodge-based.tw,kf.

35. highway-based.tw,kf.

36. (survival adj1 sex).tw,kf.

37. (compensated adj1 dating).tw,kf.

38. (prostitu* adj1 patronage).tw,kf.

39. (migrato* adj3 prostitu*).tw,kf.

40. sex tourism.tw,kf.

41. (femal* adj3 sex adj3 work*).tw,kf.

42. impersonal sex.tw,kf.

43. fsw.tw,kf.

44. transac* sex work*.tw,kf.

45. transac* sex.tw,kf.

46. (female adj3 exotic adj3 danc*).tw,kf.

47. exotic dance clu*.tw,kf.

48. Female entertainment.mp. and sex work*.tw,kf. [mp=title, abstract, original title, name of substance word, subject heading word, floating sub-heading word, keyword heading word, organism supplementary concept word, protocol supplementary concept word, rare disease supplementary concept word, unique identifier, synonyms]

49. fesw.tw,kf.

50. (street adj3 sex adj3 work*).tw,kf.

51. (street-based adj3 sex adj3 work*).tw,kf.

52. (sex adj3 work adj3 venue).tw,kf.

53. (sex adj3 exchange).tw,kf.

54. (voluntary adj3 sex adj3 work).tw,kf.

55. establishment-based.tw,kf.

56. (agency-based adj3 sex adj3 work*).tw,kf.

57. (agency-based adj3 male adj3 sex adj3 work*).tw,kf.

58. (agency-based adj3 female adj3 sex adj3 work*).tw,kf.

59. (informal adj3 sex adj3 work*).tw,kf.

60. (informal adj3 sex-work*).tw,kf.

61. (female adj3 bar adj3 work*).tw,kf.

62. information.mp. and communication tech*.tw,kf. [mp=title, abstract, original title, name of substance word, subject heading word, floating sub-heading word, keyword heading word, organism supplementary concept word, protocol supplementary concept word, rare disease supplementary concept word, unique identifier, synonyms]

63. human-computer inter*.tw,kf.

64. HCI.tw,kf.

65. computer-human inter*.tw,kf.

66. (internet or internet-based or website or web site).tw,kf.

67. online.tw,kf.

68. digital tech*.tw,kf.

69. (tablet or laptop or desktop computer or computer).tw,kf.

70. (Facebook or Twitter or WhatsApp or text messag* or social media).tw,kf.

71. (smartphon* or smart phon* or cell phon* or mobile phon*).tw,kf.

72. mobile health app*.tw,kf.

73. exp Mobile Applications/ or exp Cell Phone/

74. (smartwatch or smart watch).tw,kf.

75. (Samsung or Android).tw,kf.

76. iPhone.tw,kf.

77. exp Telemedicine/ or exp Medical Informatics/

78. "information and communication technology".tw,kf.

79. exp Cell Phone/ or exp Smartphone/

80. exp Computers, Handheld/

81. ("tablet computer" or "computer tablet" or iPad or "iPad tablet" or laptop or "desktop computer" or computer).tw,kf.

82. mobile-health.tw,kf.

83. digital technolog*.tw,kf.

84. e-health.tw,kf.

85. digital health.tw,kf.

86. mobile health.tw,kf.

87. electronic health.tw,kf.

88. smartphone app*.tw,kf.

89. mHealth.tw,kf.

90. mobile app*.tw,kf.

91. m-health.tw,kf.

92. (smart adj3 phone adj3 app*).tw,kf.

93. eHealth.tw,kf.

94. mobile phone app*.tw,kf.

95. app user*.tw,kf.

96. mHealth app*.tw,kf.

97. (mobile adj3 health adj3 app*).tw,kf.

98. (mobile adj3 phone adj3 health adj3 app*).tw,kf.

99. mobile tech* health app*.tw,kf.

100. mobile tech* app*.tw,kf.

101. (m-health adj3 app*).tw,kf.

102. telehealth.tw,kf.

103. web-based.tw,kf.

104. mobile-based.tw,kf.

105. technology platform.tw,kf.

106. health technology.tw,kf.

107. health tech*.tw,kf.

108. (health adj3 tech*).tw,kf.

109. (health adj3 app*).tw,kf.

110. text message intervention.tw,kf.

111. (text adj3 mess* adj3 inter*).tw,kf.

112. (mhealth adj3 inter*).tw,kf.

113. mobile app-based.tw,kf.

114. (mobile adj3 app* adj3 based).tw,kf.

115. mobile devic*.tw,kf.

116. mobile tech*.tw,kf.

117. mobile-health app*.tw,kf.

118. mobile health approach.tw,kf.

119. general health app*.tw,kf.

120. m-Health 2*.tw,kf.

121. Internet.mp. and communication technology.tw,kf. [mp=title, abstract, original title, name of substance word, subject heading word, floating sub-heading word, keyword heading word, organism supplementary concept word, protocol supplementary concept word, rare disease supplementary concept word, unique identifier, synonyms]

122. information technology.tw,kf.

123. (telehealth adj3 ((information and communication technology) adj3 ICT)).tw,kf.

124. technology us*.tw,kf.

125. Internet-enabled technology.tw,kf.

126. human computer inter*.tw,kf.

127. Web 2*.tw,kf.

128. Web 2* app*.tw,kf.

129. social software.tw,kf.

130. human-machine inter*.tw,kf.

131. (online adj3 support adj3 grou*).tw,kf.

132. computer us*.tw,kf.

133. (computer adj3 us*).tw,kf.

134. internet access.tw,kf.

135. internet us*.tw,kf.

136. (internet adj3 us*).tw,kf.

137. (internet adj3 access).tw,kf.

138. informatics.tw,kf.

139. cellphone.tw,kf.

140. internet resources.tw,kf.

141. (internet adj3 resources).tw,kf.

142. social media.tw,kf.

143. (social adj3 media).tw,kf.

144. instagram.tw,kf.

145. pinterest.tw,kf.

146. reddit.tw,kf.

147. smartphone-based.tw,kf.

148. phone app*.tw,kf.

149. or/1-61

150. or/62-148

151. 149 and 150
